# Myopietherapie und Prophylaxe mit „Defocus Incorporated Multiple Segments“-Brillengläsern

**DOI:** 10.1007/s00347-021-01452-y

**Published:** 2021-07-08

**Authors:** Hakan Kaymak, Birte Graff, Kai Neller, Achim Langenbucher, Berthold Seitz, Hartmut Schwahn

**Affiliations:** 1 Internationale Innovative Ophthalmochirurgie GbR, Theo-Champion-Str. 1, 40549 Düsseldorf, Deutschland; 2 Institut für Experimentelle Ophthalmologie, Universitätsklinikum des Saarlandes UKS, Homburg/Saar, Deutschland; 3 Klinik für Augenheilkunde, Universitätsklinikum des Saarlandes UKS, Homburg/Saar, Deutschland

**Keywords:** Achslänge, Augengesundheit, DIMS, Kinder, Peripherer Defokus, Axial length, Children, DIMS, Eye health, Peripheral defocus

## Abstract

Ein übermäßiges Längenwachstum des Auges bei Kindern und Jugendlichen führt zu progredienter Myopie, die im Erwachsenenalter schwerwiegende Augenerkrankungen zur Folge haben kann. Es wurden bereits verschiedene Strategien zur Hemmung der Myopieprogression entwickelt. Das vorgestellte neuartige Einstärkenbrillenglas mit „Defocus Incorporated Multiple Segments (DIMS)“-Technologie erweitert das Portfolio der Myopietherapien um eine leicht anzuwendende nichtinvasive Option. Erste Studien dazu liefern vielversprechende Ergebnisse bei sehr geringem Nebenwirkungsprofil.

## Abstract (en)

Excessive axial eye growth in children and adolescents leads to progressive myopia and can result in severe ocular diseases in adulthood. Various strategies have already been developed to inhibit progression of myopia. The novel single vision lens presented in this article features the defocus incorporated multiple segments (DIMS) technology and adds an easy to use, noninvasive option to the portfolio of myopia treatment. Initial studies showed promising results with only a very low side effect profile.

## Hintergrund

Myopie (Kurzsichtigkeit) führt zu unscharfem Sehen in der Ferne. Durch ein Missverhältnis zwischen Brechkraft und Achslänge des Auges liegt der Fernbrennpunkt nicht auf, sondern vor der Netzhaut im Glaskörper. Am häufigsten ist dieses Missverhältnis auf einen zu langen Bulbus (Achsenmyopie) zurückzuführen. Die Myopie beginnt meist im Kindesalter [36], typischerweise nach Eintritt in die Grundschule und zeigt während des Teenageralters eine Progression, die bis ins junge Erwachsenenalter fortschreiten kann. Mit zunehmender Achslänge des Auges steigt das Risiko für schwerwiegendere Erkrankungen wie Glaukom, Netzhautablösung und choroidale Neovaskularisation [11, 26, 34]. Schätzungen zufolge könnten bis 2050 10 % der Weltbevölkerung hoch myop (mehr als −5 dpt) sein, verbunden mit einem vielfachen Risiko einer solchen schwerwiegenden Erkrankung [12]. Auch in Europa wurde ein Anstieg der Myopieprävalenz beobachtet [32], besonders in der Gruppe der jungen Erwachsenen [33]. So gilt die Myopie mittlerweile als eine wesentliche Belastung für das Gesundheitswesen [1]. Es wurden Strategien entwickelt, um die Progression der Myopie zu hemmen („Myopietherapie“): die pharmakologische Therapie mit Atropin, Orthokeratologiekontaktlinsen („Ortho-K“), formstabile oder weiche multifokale Kontaktlinsen sowie Bifokal- oder Gleitsichtbrillengläser [1]. Neuartige Brillengläser mit peripher defokussierender Funktion, der sog. *D*efocus *I*ncorporated *M*ultiple *S*egments (DIMS)-Technologie sind seit April 2021 von Hoya unter dem Handelsnamen „MiYOSMART“ (Hoya Lens Thailand Ltd., Bangkok, Thailand) auch in Deutschland, Österreich und der Schweiz verfügbar.

## Wirkprinzip

**Figure 1:**
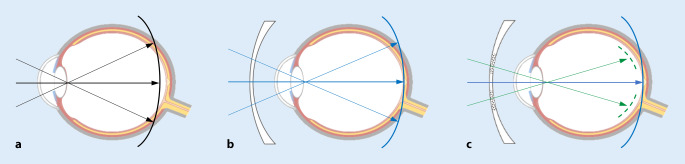

Aus Tierversuchen ist bekannt, dass ein durch Plusgläser induzierter myoper Defokus auf der Netzhaut (Fokus vor der Netzhaut im Glaskörper) das Augenlängenwachstum hemmt, während ein durch Minusgläser induzierter hyperoper Defokus (Fokus hinter der Netzhaut) das Wachstum fördert [30]. Durch Korrektion mit herkömmlichen Brillengläsern wird die „Fokusebene“, die sog. Bildschale, ferner Objekte beim myopen Auge zentral zwar auf die Makula verschoben, in dem überwiegenden Teil der Netzhaut, den perifovealen und peripheren Netzhautabschnitten liegt sie dann jedoch hinter der Netzhaut [21]. Es entsteht so über einen großen Bereich ein hyperoper Defokus (Abb. 1b), wodurch das Augenlängenwachstum angeregt werden kann. Die gezielte myope Defokussierung gerade in der Netzhautperipherie, wie in Abb. 1c dargestellt, hemmt hingegen in zahlreichen Tierversuchen das Augenlängenwachstum [2, 3, 6, 22]. Das funktioniert auch dann, wenn der myope Defokus zusätzlich zum scharfen Netzhautbild in der Makula angeboten wird. Die gezielte myope Defokussierung in der peripheren Netzhaut kann das übermäßige Augenlängenwachstum myoper Kinder verlangsamen und die Myopieprogression effektiv hemmen [7, 9].

Bei den DIMS-Gläsern wird dieser zusätzliche periphere myope Defokus mittels 396 kleiner Linsen erzeugt, die auf der Vorderfläche des Einstärkenglases eingelassen sind. Sie bilden einen ringförmigen Bereich („DIMS-Bereich“) um einen freien zentralen Bereich, dessen Durchmesser in der aktuellen praktischen Umsetzung der MiYOSMART-Gläser 9,4 mm beträgt. Das Sehen bei Blick durch den zentralen Bereich bleibt unbeeinflusst und entspricht dem Sehen mit Einstärkengläsern. Der Außendurchmesser des ringförmigen DIMS-Bereichs beträgt 33 mm. Jede einzelne defokussierende Linse hat einen Durchmesser von 1,03 mm und vermittelt jeweils einen myopen Defokus von +3,5 dpt [18, 31].

**Figure 2:**
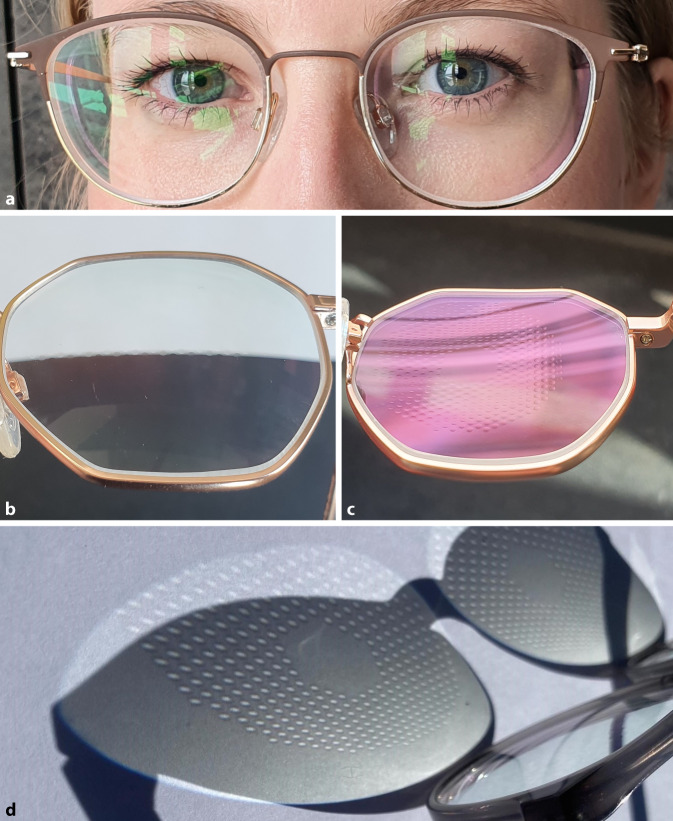

Anders als bei Gleitsicht- oder Bifokalgläsern, welche mit einer zusätzlichen Nahaddition im herkömmlichen Sinne arbeiten, bilden die Linsen der DIMS-Gläser einzelne myope Defokussierungen, die sich nicht zu einer zusammenhängenden zweiten Bildschale zusammensetzen [13]. Die DIMS-Gläser enthalten daher keine Nahaddition, die zum Lesen genutzt werden kann. Da kein gradueller Stärkenanstieg wie bei einem progressiven Glas vorliegt, kommt es bei den DIMS-Gläsern nicht zu unerwünschten Astigmatismen verschiedener Achslagen, die einen wahrgenommenen „Schaukeleffekt“ erzeugen. Die kleinen Linsen sind kosmetisch sehr unauffällig: Ein Gegenüber sieht der Brille kaum an, dass es sich um spezielle Gläser handelt (Abb. 2).

## Diagnostik und Indikationsstellung

Für die Myopiekontrolle ist neben der Bestimmung der Refraktion unter Zykloplegie, v. a. die genaue Messung der Achslänge unerlässlich. Anhand der Achslänge kann die Entwicklung einer Myopie bereits im Entstehen entdeckt werden, noch bevor eine messbare myope Refraktion vorliegt [24, 25]. Es hat sich bisher bewährt, die ermittelte Achslänge in die Perzentilenkurven von Tideman et al. [27] oder Ähnliche einzutragen, um so das individuelle Risiko der Entwicklung einer (hohen) Myopie abzuschätzen. So kann sichergestellt werden, dass eine Achsenmyopie und keine reine Brechungsmyopie vorliegt. Nur bei der Achsenmyopie kann eine das übermäßige Augenlängenwachstum hemmende Therapie helfen.

Generell können die DIMS-Gläser bei allen Kindern Anwendung finden, die eine Brille als alltägliches Korrektionsmittel akzeptieren. Der Lieferbereich der Hoya MiYOSMART-Gläser reicht sphärisch von plan bis −10 dpt und zylindrisch bis −4 dpt (maximal −10 dpt im stärksten Hauptschnitt). Da die Brillengläser exakt zentriert werden müssen, erscheinen Kinder mit Strabismus nicht geeignet, auch wenn prismatische Gläser (mit bis zu 3 pdpt) ebenfalls verfügbar sind.

## Bisherige Studienergebnisse

**Figure 3:**
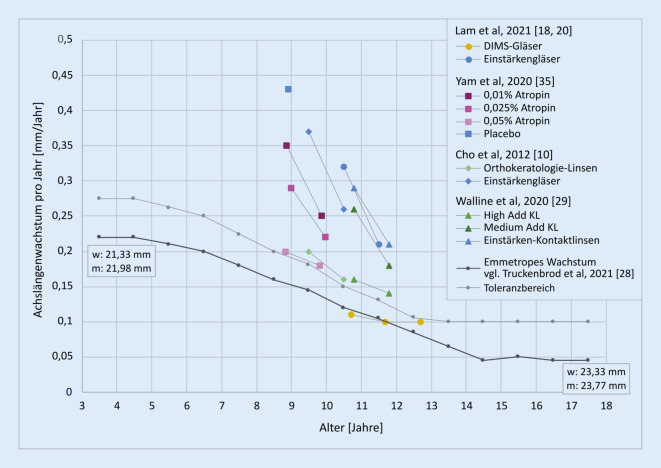

Evaluiert wurden die DIMS-Gläser in der Gruppe um Carly Siu-Yim Lam und Chi-Ho To an der Hong Kong Polytechnic University (Patente in China, Hongkong und USA: CN 10678572 B, US 10,268,050 B2). Ergebnisse einer kontrollierten Studie an myopen chinesischen Schulkindern (DIMS-Gruppe: *n* = 79, −2,97 ± 0,97 dpt, 10,20 ± 1,47 Jahre, Kontrollgruppe: *n* = 81, −2,76 ± 0,96 dpt, 10,00 ± 1,45 Jahre) sind verfügbar [18]: Nach 24 Monaten Therapie konnte im Vergleich zu herkömmlichen Einstärkengläsern eine Hemmung der Myopieprogression um 0,55 dpt (Hemmung 59 %) und des Augenlängenwachstums um 0,32 mm (Hemmung 60 %) beobachtet werden, ohne messbare Einschränkungen in Visus und Akkommodation. Die unbehandelten myopen Kinder wiesen ein typisch erhöhtes Wachstum von ca. 0,3 mm/Jahr im ersten Jahr und 0,2 mm/Jahr im zweiten Jahr auf. Die Kinder der DIMS-Gruppe hingegen hatten ein Augenlängenwachstum von durchschnittlich nur 0,1 mm/Jahr. Ein vergleichbares Wachstum von etwa 0,1 mm/Jahr wird bei gleichaltrigen kaukasischen Kindern beobachtet, welche dann im Alter von 18 Jahren mit 23,33 mm (weiblich) und 23,77 mm (männlich) eine Achslänge besitzen, die mit Emmetropie assoziiert wird [28]. Die Abb. 3 zeigt eine aus den epidemiologischen Wachstumsdaten von Truckenbrod et al. [28] ermittelte und mit eigenen epidemiologischen Erhebungen an deutschen Schulkindern [16, 17] verifizierte Kurve der Rate des Augenlängenwachstums von emmetropen Kindern. Zusätzlich sind in Abb. 3 die aus aktuellen Studiendaten ermittelten mittleren Wachstumsraten der Studien zu den DIMS-Gläsern [18, 20], aus der LAMP-Studie (Yam et al. 2020) zur Wirkung von Atropin-Augentropfen auf die Myopieprogression [35], aus der ROMIO-Studie (Cho et al. 2012) zur Wirkung von Orthokeratologielinsen [10] und aus der BLINK-Studie (Walline et al. 2020) zur Wirkung von multifokalen Kontaktlinsen [29] eingezeichnet.

## Akzeptanz und mögliche Nachteile

Die Akzeptanz der DIMS-Brillen wurde ebenfalls an chinesischen Kindern evaluiert [23]: Mit dem Wissen, dass die DIMS-Gläser die Myopieprogression hemmen können, würden sich 90 % der Kinder für die DIMS-Gläser entscheiden. Korrekte Zentrierung vorausgesetzt, entspricht der Visus beim Blick geradeaus durch die DIMS-Gläser (freier zentraler Bereich) dem Visus mit herkömmlichen Einstärkengläsern. Beim Blick durch den DIMS-Bereich der Gläser (Blickauslenkung nach nasal, temporal, oben oder unten) ist ein Visusabfall von durchschnittlich 0,06 logMAR zu beobachten, der auch nach 1 Woche des Tragens der DIMS-Gläser bestehen bleibt. Als Hauptbeschwerde wurde eine verschwommene Sicht beim Blick durch den DIMS-Bereich des Glases angegeben, welche von den Befragten allerdings nur 1‑ oder 2‑mal am Tag bemerkt wurde [23]. Eine nachteilige Gewöhnung an die DIMS-Gläser wurde nicht beobachtet. Auch nach längerem Tragen und Wechsel zurück auf Einstärkengläser traten keine Einschränkungen im Fern- und Nahvisus bei hohem oder niedrigem Kontrast sowie bei Akkommodation und Stereosehschärfe auf [19].

## Therapiebeginn

Bei der Myopietherapie ist eine umfangreiche Aufklärung über die therapeutische Wirkung wichtig für Therapietreue und -erfolg. Um eine unrealistische Erwartung der Beteiligten (Eltern und Kinder) zu vermeiden, sollte dabei im Vordergrund stehen, dass das primäre und langfristige Ziel die Hemmung der Myopieprogression ist. Ein Stillstand der Myopie wäre ein überragendes Ergebnis; ein Rückgang der Myopie ist nicht zu erwarten.

Wichtig ist es außerdem zu verdeutlichen, dass die Therapie konsequent durchgeführt werden muss, um den maximal möglichen Effekt zu erzielen. In den Studien wurden die DIMS-Brillen im Schnitt 15 h am Tag getragen. Dieselbe Tragedauer ist zu fordern, wenn ähnlich signifikante Therapieeffekte erreicht werden sollen. Da die DIMS-Brille wie eine gewöhnliche Brille mit Einstärkengläsern getragen wird, können solche Tragezeiten auch erwartet werden.

Bei der Anpassung der DIMS-Gläser ist insbesondere eine korrekte Zentrierung wichtig, damit die Akzeptanz der Brille nicht dadurch reduziert wird, dass das Kind etwa schon beim Blick geradeaus durch den defokussierenden DIMS-Bereich schaut. Es ist daher eine enge Zusammenarbeit mit einem geschulten Optiker nötig. Die Herstellerin bietet dazu spezielle Einschleifanweisungen für Optiker an.

Der Text auf der Verordnung könnte z. B. lauten „DIMS-Gläser zur Hemmung der Myopieprogression“. Die Kosten der Myopietherapie mit DIMS-Brille belaufen sich aktuell auf ca. 280 € pro Glas. Damit liegen die Gesamtkosten im Bereich dessen, was für andere bekannte Therapieoptionen investiert werden muss. Die Kosten für eine herkömmliche Einstärkenbrille entfallen, da diese ja direkt und vollständig durch die DIMS-Brille ersetzt wird.

## Therapiekontrolle und zusätzliche Therapieoptionen

Die Autoren empfehlen, die Kinder 1 bis 2 Wochen nach Erstversorgung mit der DIMS-Brille zu kontaktieren, um die aktuelle Verträglichkeit der Gläser zu erfragen.

Im weiteren Verlauf sind halbjährliche Kontrollen zu empfehlen; zunächst um zu überprüfen, ob die Stärke der DIMS-Brille und die aktuell ermittelte Refraktion noch übereinstimmen. Führt eine Stärkenänderung von ≥0,5 dpt in Richtung Myopie zu einem Anstieg des Fernvisus, ist es empfehlenswert, die Gläser anzugleichen. Geschieht dies bereits innerhalb von 12 Monaten, werden die Kosten für neue MiYOSMART-Brillengläser von der Herstellerin übernommen. Zusätzlich zur Refraktion ist bei der Therapiekontrolle auch die regelmäßige und exakte Messung der Achslänge unerlässlich. Durch das Eintragen dieser Werte in die Perzentilenkurven von Tideman et al. [27] oder Truckenbrod et al. [28] kann der Verlauf des individuellen Myopierisikos und damit die Wirksamkeit der Myopietherapie beurteilt werden.

## Anwendung in der Praxis

Bis zum Alter von 13 Jahren kann ein Augenlängenwachstum von nicht mehr als 25 % über dem Wachstum eines entsprechenden emmetropen Auges (s. Abb. 3) als Therapieziel akzeptiert werden. Da die Wachstumsrate ab etwa dem 13. Lebensjahr fast konstant bleibt, soll die Grenze des noch akzeptablen Augenlängenwachstums ab 13 Jahren bei nicht mehr als 0,1 mm/Jahr festgelegt werden. Innerhalb dieser Grenzen würde die verbleibende Progression einer weiteren Zunahme der Myopie bis zum Erwachsenenalter um nur noch ca. 1–1,5 dpt entsprechen. Es gibt bezüglich der Myopietherapie mit Brillengläsern in Deutschland noch keine offizielle Leitlinie, weswegen die oben genannte Vorgehensweise zu Therapie und Versorgung lediglich die aktuelle Einschätzung der Autoren beschreibt.

## Alternative zu bekannten Therapieoptionen?

Gemäß Abb. 3, nach aktueller Studienlage, erfüllt allein die DIMS-Brille im Beobachtungszeitraum [18, 20] das hier vorgestellte praktische Therapiekriterium vollständig, während die Therapie mit 0,05 % Atropin [35] und danach die Ortho-K-Therapie [10] und die Therapie mit „High Add“-multifokalen Kontaktlinsen [29] eine noch tolerierbare Therapieperformance erreichen können. Wie aber kürzlich von Joachimsen et al. berichtet [14], müsse eine Atropin-Konzentration von 0,05 % bei kaukasischen Kindern aufgrund der Nebenwirkungen in der praktischen klinischen Anwendung als kritisch eingestuft werden. Niedrigere Atropin-Konzentrationen (0,01 und 0,025 %), die zurzeit in Deutschland als First-Line-Therapie angedacht sind [8], waren nach der Auswertung gemäß Abb. 3 und nach eigenen Untersuchungen der Autoren [15] deutlich weniger wirksam.

Die kürzlich veröffentlichten 1‑ und 2‑Jahres-Ergebnisse des ähnlich konstruierten Brillenglases „Stellest“ der Firma Essilor zeigten ebenfalls eine effektive Hemmung der Myopieprogression und ein geringes jährliches Augenlängenwachstum [4, 5], besonders bei einer Tragedauer ≥12 h/Tag.

## Pharmakologische Zusatztherapie?

Zu der Kombination von DIMS-Brillengläsern und Atropin gibt es zurzeit noch keine systematischen Untersuchungen. Dass die Atropin-Therapie die therapeutische Wirkung der DIMS-Gläser verstärkt oder ergänzt und die therapeutische Lücke dadurch geschlossen werden kann, ist bisher Spekulation. Wird mit der DIMS-Brille allein schon das Therapieziel erreicht, könnte auf eine aufwendigere Zusatztherapie mit Atropin verzichtet werden. Liegt das mittels DIMS-Gläser reduzierte Augenlängenwachstum aber außerhalb des vorgeschlagenen Toleranzbereichs, kann zu einer möglichen Steigerung der Therapieeffizienz eine ergänzende pharmakologische Therapie erfolgen. Ob bekannte Nebenwirkungen des Atropins (erweitere Pupille, verringerte Akkommodation) zusätzliche Einschränkungen beim Tragen der DIMS-Gläser mit sich bringen oder ob der defokussierende DIMS-Bereich unter Atropin weniger stark wahrgenommen wird, wird von uns gegenwärtig untersucht. Anders als bei der Kombination von Bifokal- oder Gleitsichtgläsern mit Atropin kann der DIMS-Bereich nicht als Nahunterstützung zur Kompensation der Atropin-bedingt verringerten Akkommodation dienen.

## Fazit für die Praxis

- Die Myopietherapie mit Brillengläsern ist verblüffend einfach anzuwenden: Kindern könnten anstelle herkömmlicher Einstärkengläser mit DIMS-Gläsern versorgt werden, welche Myopiekorrektur und -therapie in einem bieten.
- Neben der Refraktion ist die Messung der Achslänge des Auges wichtig zur Indikationsstellung und Verlaufsbeurteilung einer Myopietherapie.
- Eine Myopietherapie sollte stets früh begonnen und konsequent durchgeführt werden.
- Das ideale Therapieziel der Myopietherapie, die Herstellung eines emmetropen Wachstums, wird laut aktuellen Studiendaten mit den DIMS-Brillengläsern erreicht.
- Studiendaten zur Wirksamkeit der DIMS-Gläser speziell bei europäischen Kindern und von anderen Forschungsgruppen stehen derzeit noch aus.

## References

[1] Ang M, Wong TY (2020). Updates on myopia. A clinical perspective.

[2] Arumugam B, Hung L-F, To C-H, Holden B, Smith EL (2014). The effects of simultaneous dual focus lenses on refractive development in infant monkeys. Invest Ophthalmol Vis Sci.

[3] Arumugam B, Hung L-F, To C-H, Sankaridurg P, Smith EL (2016). The effects of the relative strength of simultaneous competing defocus signals on emmetropization in infant rhesus monkeys. Invest Ophthalmol Vis Sci.

[4] Bao J, Huang Y, Li X, Yang A, Lim EW, Spiegel D, Drobe B, Chen H (2021) Myopia control with spectacle lenses with aspherical lenslets: a 2‑year randomized clinical trial. https://arvo2021.arvo.org/meetings/virtual/HAvS9pqaZyKj8e7i6. Zugegriffen: 25. Mai 2021

[5] Bao J, Yang A, Huang Y, Li X, Pan Y, Ding C, Lim EW, Zheng J, Spiegel DP, Drobe B, Lu F, Chen H (2021). One-year myopia control efficacy of spectacle lenses with aspherical lenslets. Br J Ophthalmol.

[6] Benavente-Pérez A, Nour A, Troilo D (2014). Axial eye growth and refractive error development can be modified by exposing the peripheral retina to relative myopic or hyperopic defocus. Invest Ophthalmol Vis Sci.

[7] Berntsen DA, Barr CD, Mutti DO, Zadnik K (2013). Peripheral defocus and myopia progression in myopic children randomly assigned to wear single vision and progressive addition lenses. Invest Ophthalmol Vis Sci.

[8] Berufsverband der Augenärzte Deutschlands, Deutsche Ophthalmologische Gesellschaft (2018). Stellungnahme des Berufsverbandes der Augenärzte Deutschlands, der Deutschen Ophthalmologischen Gesellschaft. Empfehlungen bei progredienter Myopie imd Kindes- und Jugendalter.

[9] Chakraborty R, Ostrin LA, Benavente-Pérez A, Verkicharla PK (2020). Optical mechanisms regulating emmetropisation and refractive errors: evidence from animal models. Clin Exp Optom.

[10] Cho P, Cheung S-W (2012). Retardation of myopia in orthokeratology (ROMIO) study: a 2-year randomized clinical trial. Invest Ophthalmol Vis Sci.

[11] Flitcroft DI (2012). The complex interactions of retinal, optical and environmental factors in myopia aetiology. Prog Retin Eye Res.

[12] Holden BA, Fricke TR, Wilson DA, Jong M, Naidoo KS, Sankaridurg P, Wong TY, Naduvilath TJ, Resnikoff S (2016). Global prevalence of myopia and high myopia and temporal trends from 2000 through 2050. Ophthalmology.

[13] Jaskulski M, Singh NK, Bradley A, Kollbaum PS (2020). Optical and imaging properties of a novel multi-segment spectacle lens designed to slow myopia progression. Ophthalmic Physiol Opt.

[14] Joachimsen L, Farassat N, Bleul T, Böhringer D, Lagrèze WA, Reich M (2021). Side effects of topical atropine 0.05 % compared to 0.01 % for myopia control in German school children: a pilot study. Int Ophthalmol.

[15] Kaymak H, Graff B, Schaeffel F, Langenbucher A, Seitz B, Schwahn H (2021). A retrospective analysis of the therapeutic effects of 0.01% atropine on axial length growth in children in a real-life clinical setting. Graefes Arch Clin Exp Ophthalmol.

[16] Kaymak H, Neller K, Graff B, Körgesaar K, Langenbucher A, Seitz B, Schwahn H (2021). Optometrische Schulreihenuntersuchungen. Erste epidemiologische Daten von Kindern und Jugendlichen der 5. bis 7. Klasse. Ophthalmologe.

[17] Kaymak H, Neller K, Funk S, Seitz B, Langenbucher A, Schwahn H (2021). Optometrische Schulreihenuntersuchungen. Erste Ergebnisse eines Pilotprojektes zur logistischen Machbarkeit. Ophthalmologe.

[18] Lam CSY, Tang WC, Tse DY, Lee RPK, Chun RKM, Hasegawa K, Qi H, Hatanaka T, To CH (2020). Defocus incorporated multiple segments (DIMS) spectacle lenses slow myopia progression: a 2-year randomised clinical trial. Br J Ophthalmol.

[19] Lam CSY, Tang WC, Qi H, Radhakrishnan H, Hasegawa K, To CH, Charman WN (2020). Effect of defocus incorporated multiple segments spectacle lens wear on visual function in myopic Chinese children. Transl Vis Sci Technol.

[20] Lam CS, Tang WC, Lee PH, Zhang HY, Qi H, Hasegawa K, To CH (2021). Myopia control effect of defocus incorporated multiple segments (DIMS) spectacle lens in Chinese children: results of a 3-year follow-up study. Br J Ophthalmol.

[21] Lin Z, Martinez A, Chen X, Li L, Sankaridurg P, Holden BA, Ge J (2010). Peripheral defocus with single-vision spectacle lenses in myopic children. Optom Vis Sci.

[22] Liu Y, Wildsoet C (2011). The effect of two-zone concentric bifocal spectacle lenses on refractive error development and eye growth in young chicks. Invest Ophthalmol Vis Sci.

[23] Lu Y, Lin Z, Wen L, Gao W, Pan L, Li X, Yang Z, Lan W (2020). The adaptation and acceptance of defocus incorporated multiple segment lens for Chinese children. Am J Ophthalmol.

[24] Mutti DO, Hayes JR, Mitchell GL, Jones LA, Moeschberger ML, Cotter SA, Kleinstein RN, Manny RE, Twelker JD, Zadnik K (2007). Refractive error, axial length, and relative peripheral refractive error before and after the onset of myopia. Invest Ophthalmol Vis Sci.

[25] Rozema J, Dankert S, Iribarren R, Lanca C, Saw S-M (2019). Axial growth and lens power loss at myopia onset in Singaporean children. Invest Ophthalmol Vis Sci.

[26] Saw S-M, Gazzard G, Shih-Yen EC, Chua W-H (2005). Myopia and associated pathological complications. Ophthalmic Physiol Opt.

[27] Tideman JWL, Polling JR, Vingerling JR, Jaddoe VWV, Williams C, Guggenheim JA, Klaver CCW (2018). Axial length growth and the risk of developing myopia in European children. Acta Ophthalmol.

[28] Truckenbrod C, Meigen C, Brandt M, Vogel M, Sanz Diez P, Wahl S, Jurkutat A, Kiess W (2021). Longitudinal analysis of axial length growth in a German cohort of healthy children and adolescents. Ophthalmic Physiol Opt.

[29] Walline JJ, Walker MK, Mutti DO, Jones-Jordan LA, Sinnott LT, Giannoni AG, Bickle KM, Schulle KL, Nixon A, Pierce GE, Berntsen DA (2020). Effect of high add power, medium add power, or single-vision contact lenses on myopia progression in children: the BLINK randomized clinical trial. JAMA.

[30] Wallman J, Winawer J (2004). Homeostasis of eye growth and the question of myopia. Neuron.

[31] Wesemann W (2019). Hoya MyoSmart – Ein radikal innovatives Brillenglas zur Myopiekontrolle. DOZ.

[32] Williams KM, Bertelsen G, Cumberland P (2015). Increasing prevalence of myopia in Europe and the impact of education. Ophthalmology.

[33] Williams KM, Verhoeven VJM, Cumberland P (2015). Prevalence of refractive error in Europe: the European Eye Epidemiology (E(3)) consortium. Eur J Epidemiol.

[34] Wong TY, Ferreira A, Hughes R, Carter G, Mitchell P (2014). Epidemiology and disease burden of pathologic myopia and myopic choroidal neovascularization: an evidence-based systematic review. Am J Ophthalmol.

[35] Yam JC, Li FF, Zhang X, Tang SM, Yip BHK, Kam KW, Ko ST, Young AL, Tham CC, Chen LJ, Pang CP (2020). Two-year clinical trial of the low-concentration atropine for myopia progression (LAMP) study: phase 2 report. Ophthalmology.

[36] Zadnik K, Sinnott LT, Cotter SA, Jones-Jordan LA, Kleinstein RN, Manny RE, Twelker JD, Mutti DO (2015). Prediction of juvenile-onset myopia. JAMA Ophthalmol.

